# Potential of Alpha-(α)-Solanine as a Natural Inhibitor of Fungus Causing Leaf Spot Disease in Strawberry

**DOI:** 10.3390/life13020450

**Published:** 2023-02-06

**Authors:** Ning Xu, Huan Lu, Xueqian Yi, Simin Peng, Xiaohui Huang, Yu Zhang, Changzheng He

**Affiliations:** 1 College of Horticulture, Hunan Agricultural University, Nongda Road No.1, Changsha 410128, China; 2 Institute of Hunan Edible Fungi, Shuangtang Road No. 107, Changsha 410013, China; 3 Institute of Edible Fungi, Shanghai Academy of Agricultural Sciences, No. 1000, Jinqi Road, Shanghai 201403, China; 4 Hunan Institute of Traffic Engineering, Jiefang Road No. 430, Hengyang 421200, China

**Keywords:** solanine, *Curvularia trifolii*, strawberry, antifungal, transcriptome

## Abstract

*Curvularia trifolii* is an important pathogenic fungus that causes leaf spot disease in strawberry and other crops. Increased resistance in pathogenic fungi against chemical fungicides necessitates the search for biological alternatives to control plant fungal diseases. The present study aimed to perform transcriptome and metabolome analysis of *C. trifolii* fungi. We evaluated the potential of an alkaloid, namely alpha (α)-solanine, to inhibit the growth of Curvularia under in vitro conditions. Furthermore, transcriptomic and metabolomic analysis of treated *C. trifolii* was performed to identify the differential genes and metabolites. Results revealed that treatment with α-solanine resulted in the poor growth and development of fungal spores. The transcriptome analysis revealed that 1413 genes were differentially expressed (DEGs), among which 340 unigenes were up-regulated, 100 unigenes were down-regulated, and the rest were unaffected in treated samples. Gene ontology analysis revealed that the majority of the genes were related to oxidative stress in the fungus. Additionally, using ultra-high performance liquid chromatography-tandem mass spectrometry, we identified 455 metabolites, among which the majority of metabolites were related to lipid biosynthesis. The high number of genes related to lipid biosynthesis and reactive oxygen species revealed that α-solanine causes oxidative stress in Curvularia, leading to growth inhibition, and can be potentially used as an alternative to chemical fungicides.

## 1. Introduction

Strawberry cultivation started during the 1980s in China and since then has been an important fruit crop of the country. China produces ~3.72 million tons of strawberry fruit annually [1]. A good strawberry crop requires healthy soil, fungicides, and intensive crop management practices to compete in terms of quantity and quality in the required market. Unfortunately, the strawberry crop is susceptible to several fungal pathogens that cause leaf spot diseases [2]. Moreover, fungi such as *Mycosphaerella fragariae* are known to infect strawberry crops worldwide [3].These leaf spot diseases can cause a decline in yield or the death of plants and are mainly caused by phytopathogenic fungi such as Cercospora, Curvularia, and Bipolaris genera [4]. In a recent study, a dozen fungal species (including *Curvularia trifolii*) were diagnosed from China that collectively cause strawberry crown rot disease [4]. Additionally, *Curvularia trifolii* can cause leaf spot disease in stawberry, which can ultimately lead to fruit rot [4]. Curvularia leaf spot disease in strawberry is an important fungal disease in China that spreads rapidly due to prolonged wet seasons [4]. Furthermore, the negative impact of the disease is severe under high temperature, rainfall, and humidity. *Curvularia trifolii* can cause severe leaf spot disease in numerous plant species, including turf grass, tobacco, and berseem clover [5,6,7,8]. The disease can spread with rain splashes and can cause yield losses of up to 50–100%. It can cause severe damage to leaves and the infected plants are not able to develop any seeds or fruit.

In the case of strawberries, leaf spot disease is manifested by leaf lesions and rotting fruits. Conventional methods of strawberry breeding are difficult due to the prolonged time required and ever-changing pathogen dynamics. Landraces are considered as a good source of resistant genotypes in strawberry crops. Since strawberry fruit is consumed on a large scale, fungicide sprays are not preferred by farmers due to their hazardous impact on human health [9]. 

α-solanine is a natural glycoalkaloid that is naturally present in plants of the nightshade family. Recently, it has been used as an active compound against allergies, bacterial and fungal pathogens, and different types of cancers [1,10,11]. Solanine is known as a toxic glycoalkaloid and is mainly derived from solanidine. It is mainly extracted from the leaves or tubers of potatoes and tomatoes. The purified alkaloid extract has shown strong antibacterial activities [12]. Strong antifungal activities against *Candida albicans* and *Trichophyton rubrum* have been reported for these alkaloids [13]. 

Comparative analysis of next-generation sequencing data is widely used to study the genetic architecture of hosts and pathogens. Due to the availability of de novo assembly tools and the decreasing cost of DNA and RNA sequencing, the genetic architectures of numerous organisms have been elucidated. The genus *Curvularia* is composed of 80 different fungal species, mainly infecting plants [14]. The commercial strawberry plant is an octaploid (2n = 8x= 56) with a genome size of 814.3 Mb [15]. In the case of fungal infection, the data on differentially expressed genes (DEG) from a known genome sequence indicate the cellular mechanisms involved in host–pathogen interaction [16]. For the identification of Curvularia defense-related genes, transcriptome profiling of resistant and susceptible buffalograss lines was performed, leading to the identification of about 461 DEGs between the resistant and susceptible lines during infection [17,18]. The study of metabolites is a relatively modern field in the study of systems biology. The exploration of metabolites after a specific treatment increases our understanding of downstream metabolic pathways. Recently, metabolic studies and transcriptomic studies have been conducted on a number of fungi [19,20,21]. 

In the present study, we used a natural well-known anti-fungal alkaloid (α-solanine) to inhibit the fungi causing leaf spot disease in strawberry. Our goal was to analyze the DEGs and metabolic profile of Curvularia fungi in the presence of α-solanine. Interestingly, several genes related to cell growth and differentiation showed strong differential expression. The metabolite analysis also suggested that the metabolic pathways related to lipid and cell growth were differentially regulated. Our results demonstrate that, similar to commercial fungicides, α-solanine also inhibits Curvularia fungus growth and can be used as an alternative approach to control fungal diseases in plants. 

## 2. Materials and Methods

### 2.1. Source of Fungi and α-Solanine

Infected strawberry leaves were collected from a field with visible symptoms of necrosis and fruit rotting. A small piece of infected strawberry leaf was rinsed with autoclaved distilled water. After washing, the leaf spot was inoculated in Luria Bertani liquid (LB-liquid) medium with dextrose for 5 days or until the rapid growth of mycelia was observed. The spores were collected by centrifugation at 10,000 rpm for 10 min and counted using a hemocytometer (Watson Bio Lab 177-112C, Kobe, Japan). The suspension for a single spore of *Curvularia trifolii* was used for the preparation of culture as described previously [22]. The optical density (OD) was measured at 600 nm (OD_600_) with a spectrophotometer (Thermo NantoDrop Lite, Madison, WI, USA). The suspension culture was prepared at a concentration of 1 × 10^6^ spores per ml. To evaluate the impact of α-solanine, a 50 mL suspension was prepared (OD_600_ = 0.5) and divided into 6 culture tubes. The α-solanine was first dissolved in dimethyl sulfoxide (DMSO), with a final concentration of 10 mg/mL. After culturing for 12 h at 28 °C, α-solanine was added to a final concentration of 5 mg/mL in 5 mL of liquid culture. After 12 h of incubation, the solution was dropped onto a hemocytometer, and spore germination was observed under a light microscope (400×) as described previously [4]. The inhibition rate of spore germination was calculated by the following formula [23].

Inhibition rate of spore germination (%) = number of ungerminated spores/total number of spores

### 2.2. RNA Extraction and Illumina Sequencing

Total RNA from both the control (designated as A) and α-solanine-treated (designated as B) samples was isolated as described previously (plant RNA purification reagent, Invitrogen, Waltham, MA, USA) [24]. The quality and integrity of total RNA was checked by running the samples on 1% agarose gel and then confirmed using a NanoDrop 2000C (Thermo Scientific, Waltham, MA, USA). Approximately 5 μg of total RNA was used for cDNA library construction according to the established protocol of the NEBNext Ultra™ RNA Library Prep Kit for illumina sequencing [24,25]. Messenger RNA was enriched by promagnetic oligo-dT beads. The first-strand cDNAs were converted into blunt end fragments before the being adenylated at the 3′ end for adapter ligation (NEB, Ipswich, MA, USA). The total samples were sequenced through the Illumina HiSeq platform. 

### 2.3. Sequence Analysis and Mapping

The sequencing data of RNA transcripts and unigenes generated in this experiment were submitted to the NCBI database and are available under the project number PRJNA885213. The transcriptomic data in this study were compared to the already existing reference genome of *Curvularia trifolii* isolated from tobacco [26]. For de novo assembly, the sequencing reads were first cleaned by using the Fastp package (https://github.com/OpenGene/fastp, accessed on 30 June 2022). The low-quality end sequences were trimmed with the Fast-X toolkit (version 0.0.14, http://hannonlab.cshl.edu/fastx_toolkit/ accessed on 30 June 2022). The quality of sequencing reads was checked and established through Sickle and SeqPrep (https://github.com/najoshi/sickle accessed on 30 June 2022). Short RNA sequencing reads were assembled using the Trinity assembler software [27]. Gene redundancy and their origin were estimated through BUSCO [28]. The good-quality RNA reads were aligned with the reference genome to accurately estimate protein structures and locations [29]. The assembled sequencing reads were subjected to BLASTX searches against the NR and Swiss-Prot protein databases to evaluate differential gene expression as described previously [30]. 

### 2.4. Gene Annotation, Enrichment, and Expression Analysis

For each category of samples, both transcripts and unigene abundance were first analyzed using TransDecoder (http://transdecoder.github.io/ accessed on 30 June 2022). The read counts were adjusted according to edgeR [31]. The values of differential gene expression were calculated by the DEGSeq R package [32]. The DEGs were functionally annotated and enriched by using the R package of GoSeq and KEGG, NR, Swiss-Prot, BLAST, COG, and PFam [33,34].

### 2.5. Metabolome Analysis of Curvularia

In order to determine the important small molecules or compounds in the *Curvularia* fungi, we used ultra-high performance liquid chromatography-tandem mass spectrometry (UHPLC-MS) as a standardized protocol using the Thermo Scientific Vanquish UHPLC system (Thermo Fisher Scientific, Waltham, MA, USA) as described previously [19,35]. Briefly, both the control and α-solanine-treated fungi were harvested at optimum OD_600_. The fungus was grown on media with an optimized vitamin concentration as described earlier [36]. The cells were harvested and washed twice with water. Equal amounts of (100 mg) cells were taken for homogenization in ice-cold methanol (Phan and Blank, 2020). Following the vacuum drying of pellets, residues were dissolved and the supernatant was passed through the 0.22 μm filter for liquid chromatography detection. Relative standard deviation (RSD) was calculated for all the peaks in LC-MS analysis. For evaluation and monitoring of quality data, the same amount of samples were processed as quality control samples (QC). 

In order to identify the differential metabolites, multivariate statistical analysis was performed, which included partial least squares-discriminant analysis (PLSDA) and principal component analysis. Additionally, R software was used to establish the relationship of metabolites with the samples. Using the KEGG pathway and human metabolome database (HMDB) analysis, functional analysis of metabolites was performed as described previously [37,38].

## 3. Results

### 3.1. Effects of α-Solanine on Spore Germination

We used light microscopy to understand the impact of α-solanine on the growth of *Curvularia trifolii* isolated from strawberry leaves. Results revealed that α-solanine treatment significantly decreased the number of fungal spores (Figure S1). It was clear that α-solanine had a negative effect on the growth of *C. trifolii* spores (Figure S1). A closer examination revealed that the spores of *C. trifolii* without treatment were complete, slender, and uniform, and the surface of the spores was smooth (Figure S1A). After treatment with α-solanine, spores of *C. trifolii* exhibited obvious changes in morphology, and some of the spores were cracked, shrunken, or had swelled, which indicated that solanine treatment affected the germination of spores (Figure S1B).

### 3.2. Evaluation of Sequence Reads and Features

We sequenced the transcriptome of control and α-solanine-treated *Curvularia* in triplicate (A1, A2, and A3 for control and B1, B2, and B3 for α-solanine-treated samples) through illumina sequencing and analyzed the general features of the sequence reads (Table 1). The total number of clean reads and their corresponding length is presented in Table 1. Total genome size coverage was 38.9 Mbp and the average N50 length was 2789 bp, with a 78% mean mapped ratio (Table S1). RNA-Seq analysis of *Curvularia* resulted in total reads of 24,213 transcripts, where 17,856 genes were unique. The longest transcript length was 16,387 bp, and the smallest reads were 201 bp (Figure S2). The average length of transcripts was recorded as 1608 bp, and the average length of unique genes was recorded as 1368 bp (with an average N50 length of 2453 bp for unigenes, as shown in Table S1). The average TransRate obtained from de novo assembly was less than 0.3% in all the samples (Table S1). The complete sequence was submitted to the NCBI under the project ID PRJNA885213.

### 3.3. Gene Annotation of Curvularia trifolii

**Figure 1:**
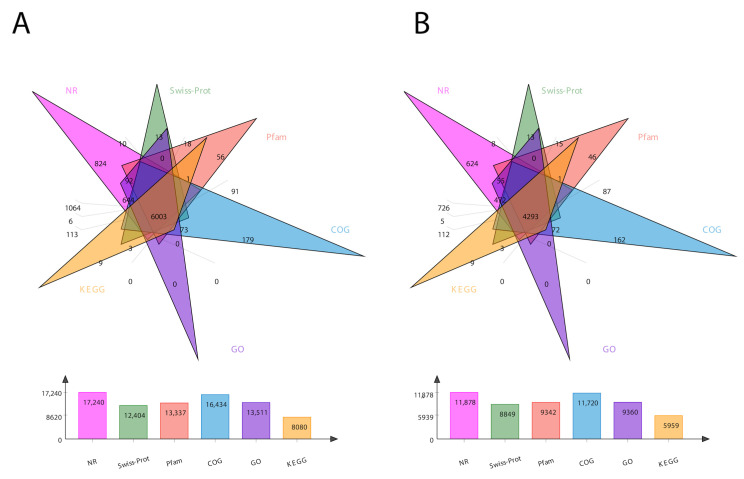
Venn diagram showing the number of DEGs in six different databases. (**A**) represents transcript data, while (**B**) represents unigene data. The number of DEGs identified by each database is presented at the bottom of each panel.

For the comprehensive analysis of transcripts, we annotated the sequencing data for both the transcripts and unigenes in six different databases, including COG, GO, KEGG, NR, Pfam, and Swiss-Prot. The total number of annotated genes for each database is presented in Figure 1. In total, 24,213 transcripts and 17,853 unigenes were annotated (Figure 1). The Venn diagram of total transcripts and unigenes was obtained according to these six protein databases (Figure 1). It shows 6003 mutual genes for all transcript data (Figure 1A), accounting for 25% of total expressed genes. For unigenes, it shows 4239 mutual genes (Figure 1B), accounting for 24% of the expressed genes. 

**Figure 2:**
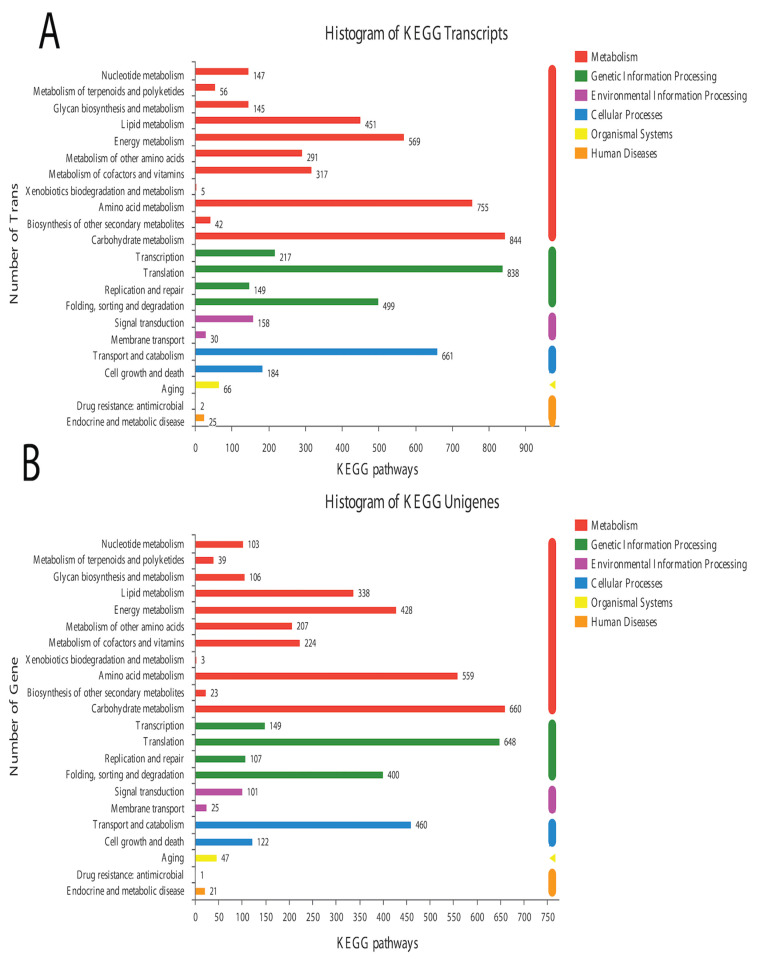
Historgram based on the total number of transcripts (**A**) and unigenes from *Curvularia trifolii* isolated from strawberry leaves. (**B**) The identified genes from transcript data can be classified into six broad groups through KEGG analysis.

In the BLAST search for transcripts and unigenes against the non-redundant database (Nr, the de novo annotations), 70% of genes shared a high similarity index (80%, Figure S3). It is obvious from the Venn diagram that the highest number of proteins was functionally annotated (17,240 and 11,878 in all transcripts and unigenes, respectively) by the NR database, validating the most identities with full-length proteins in the database. Additionally, 30% of unigenes showed more than 80% homology in the Swiss-Prot database. The second highest number of protein identities was revealed by the COGs database, which provided the greatest coverage of *Curvularia* orthologs in the database. The histogram based on the Kyoto Encyclopedia of Genes and Genomes (KEGG) resulted in a total of 6451 transcripts and 4771 unigenes, which were classified into six main functional groups (Figure 2). These six groups were categorized as genes related to metabolism, DNA/RNA regulation inside the cell, cell signaling, growth, aging, and drug resistance (Figure 2).

### 3.4. Effect of α-Solanine Treatment on the Curvularia Transcriptome

**Figure 3:**
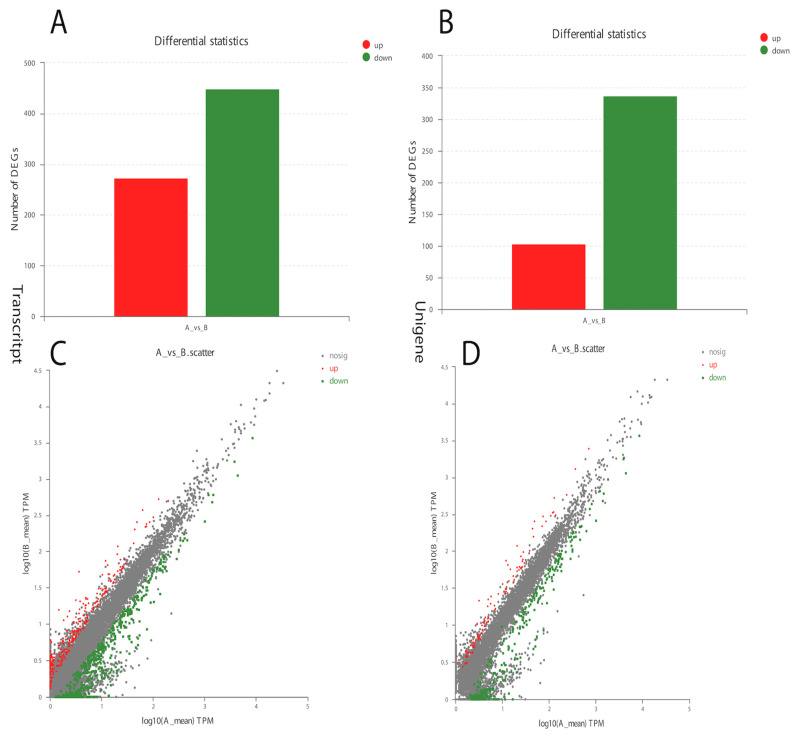
Differential gene expression in α-solanine-treated *Curvularia* fungi. (**A**). Number of transcript DEGs in the control and treated samples. (**B**). Number of up-regulated and down-regulated unigene DEGs. (**C**,**D**) represent up-regulated and down-regulated DEGs.

The analysis of *Curvularia* transcripts from treated samples detected a very good response to α-solanine treatment. A significant number of differentially expressed genes in the treated versus control *Curvularia* samples were found, indicating a higher level of changes in its transcriptome (Figure 3). Overall, in the treated *Curvularia* sequencing data, 1413 unique genes were expressed, while in the control samples 3904 unique genes were expressed (Figure S4). The analysis of DEGs revealed that, in the treated samples, 441 transcripts were down-regulated while 280 transcripts were up-regulated (Figure 3A). Likewise, 340 unigenes were down-regulated while 100 unigenes were up-regulated (Figure 3B).

**Figure 4:**
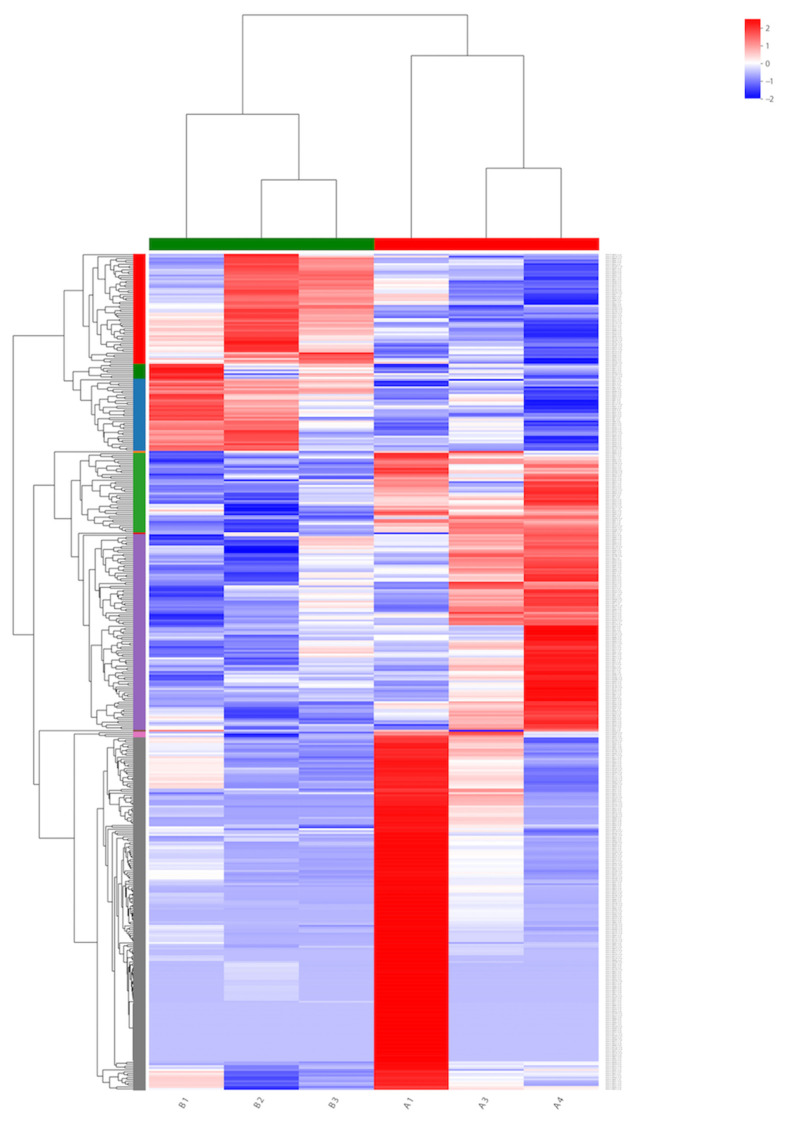
Heat map for transcripts in the treated (B1, B2, and B3) and untreated fungi transcripts (A1, A3, and A4).

To identify the genes expressed during α-solanine treatment and in the absence of any inhibitor, a Venn diagram from differentially expressed genes was generated. Based on the DEG data, a hierarchical cluster analysis (heat map analysis) was performed between α-solanine-treated and control samples (Figure 4). From the heat map analysis, it is clear that α-solanine treatment tremendously changed the gene expression profile of *Curvularia trifolii*. 

### 3.5. COG, KEGG, and GO Enrichment and Annotations for α-Solanine Responsive DEGs 

**Figure 5:**
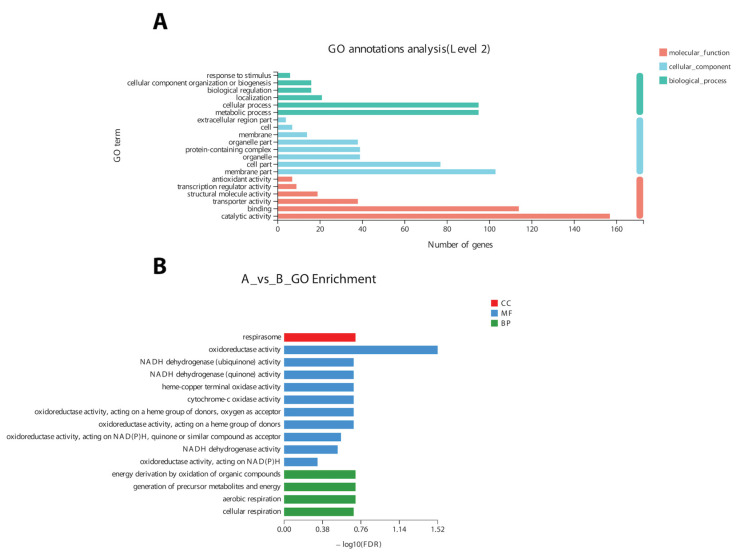
Annotation (**A**) and enrichment of DEGs (**B**) into three broad categories (molecular function (MF), cellular component (CC), and biological process(BP)) on the basis of a gene ontology search.

In the COG classification, 20 different functional annotations were categorized. Nonetheless, the majority (219 genes) of DEGs were of unknown function. Overall, most of the genes related to ribosomes (energy production and conversion) and RNA processing and cell signaling were differentially expressed. The GO annotation results revealed that DEGs (n = 929) could be divided into three broad categories (Figure 5A), i.e., molecular function (MF), cellular component (CC), and biological process (BP). 

Based on GO annotation results, changes in important biological processes were identified where the maximum number of genes was related to oxidoreductase activity and cellular respiration. The GO enrichment analysis of 9425 DEGs validated the annotation results regarding the genes related to oxidoreductase activity and cellular respiration (Figure 5B).

**Figure 6:**
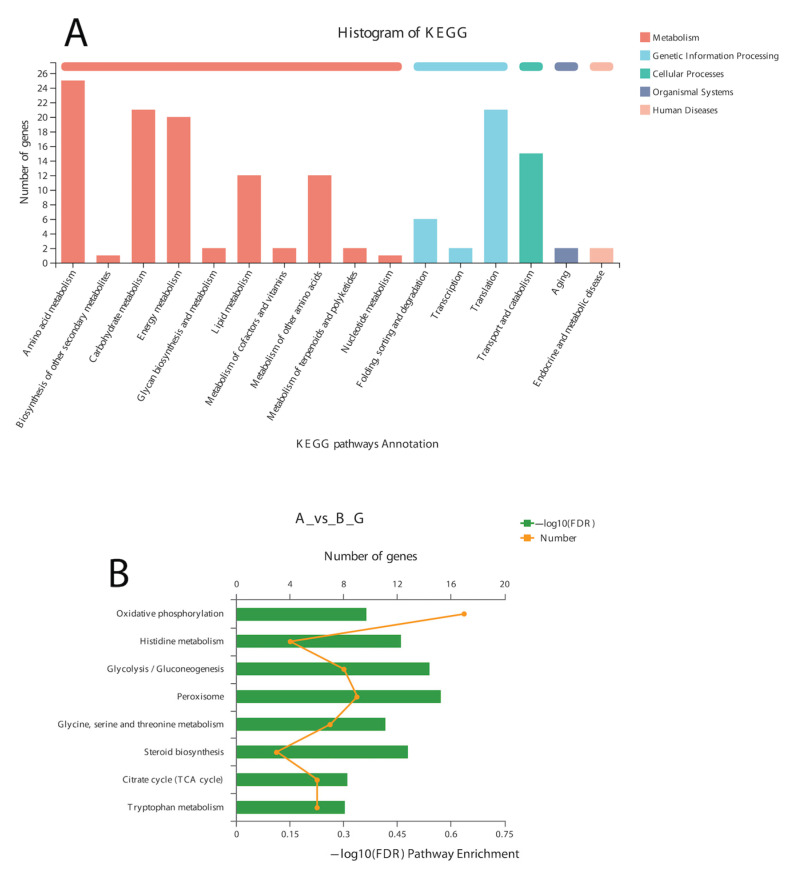
KEGG pathway annotation (**A**) and enrichment analysis (**B**) of DEGs after α-solanine treatment against *Curvularia* fungi.

A total of 12 up-regulated and 198 down-regulated genes were identified through the KEGG annotation pathway (Figure 6). The KEGG annotation of up-regulated genes identified the high expression of genes related to amino acid, carbohydrate, energy, and lipid metabolism. Furthermore, the genes related to cellular growth and aging were also up-regulated, while the genes related to metabolism, nucleic acid processing, protein folding and transport, and aging were down-regulated (Figure 6). KEGG-based gene enrichment analysis revealed eight different pathways that were differentially expressed during α-solanine treatment. These pathways included oxidative phosphorylation, histidine, tryptophan, glycine, threonine and serine metabolism, peroxisomes and steroid biosynthesis, and the citric acid cycle.

### 3.6. Effect of α-Solanine on the Metabolites of Curvularia

**Figure 7:**
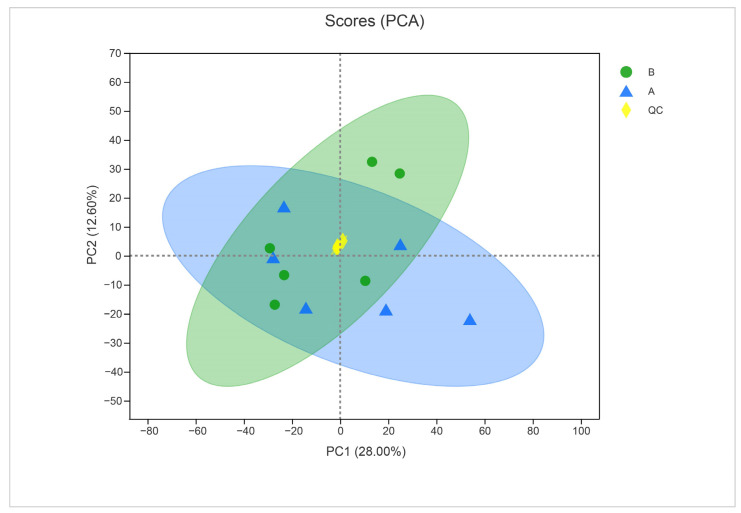
Principal component analysis of control (sample A) and α-solanine-treated *Curvularia* fungi (B) in the negative ion mode. QC represents quality control.

To ensure the quality of acquired metabolite data, we used PCA to validate the results. The close clustering of QC samples in the PCA plot indicated the reliable quality of the data (Figure 7). The two principal component analyses of metabolites from treated and untreated samples indicated a clear difference between metabolites (28% for PC1 and 12.60% for PC2).

**Figure 8:**
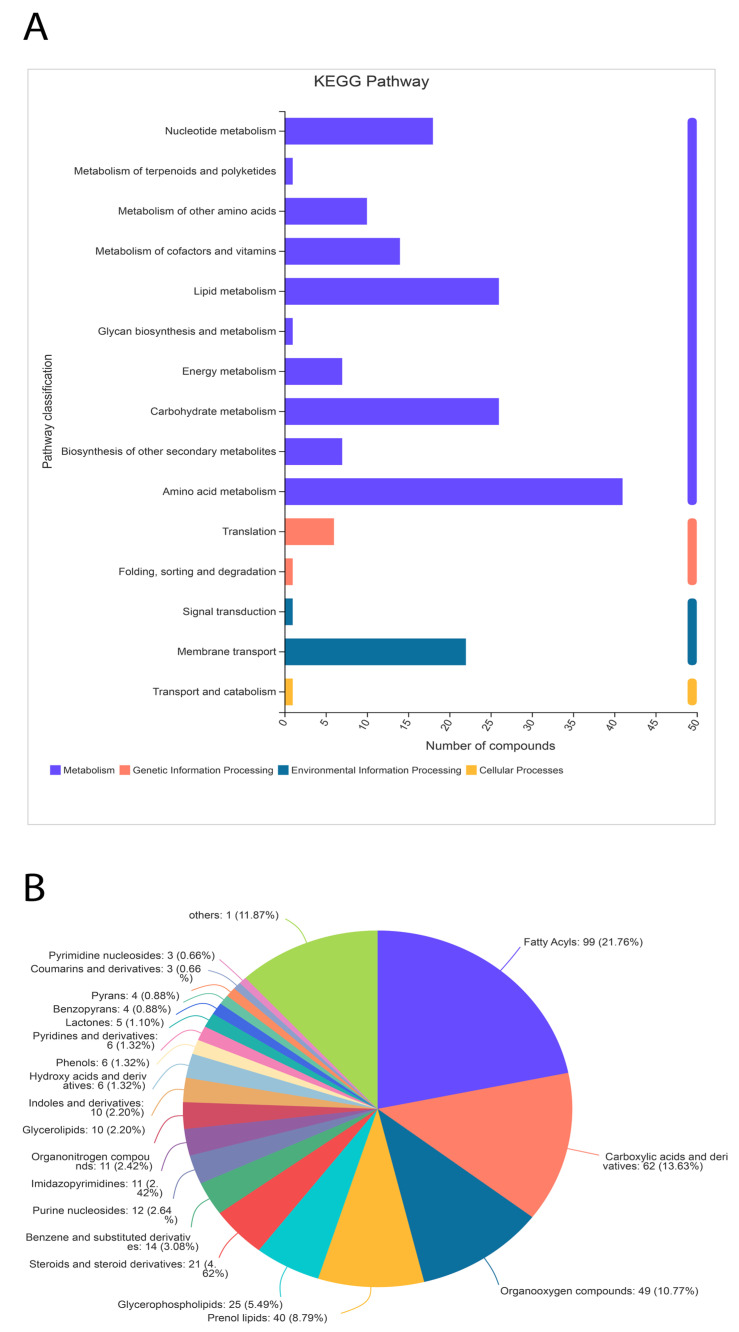
Classification of metabolites through the KEGG pathway (**A**) and HDMB (**B**) analysis. The metabolites related to lipid metabolism are highly expressed in the α-solanine-treated *Curvularia* fungi.

Significant differences between metabolites in the treated samples were observed. A total of 455 metabolites were identified through the HDMB database, which were classified into 21 different categories (Figure 8). Nonetheless, the majority (40%) of metabolites were related to lipid metabolism (196 out of 455). The second most populated category of metabolites was related to organic acids and their derivatives (Figure 8). Additionally, 10% of metabolites were identified as organic oxygen compounds.

A total of 405 metabolites were identified in the KEGG pathway analysis under four major categories (cellular metabolism, genetic and environmental factors, and cellular processing). Metabolites related to nucleotides, carbohydrates, vitamins, lipids, and other secondary metabolites were identified. However, a small amount of metabolites related to translation, signal transduction, and transport were also identified. Collectively, the HDMB and KEGG pathway data identified the most metabolites related to lipid biosynthesis.

## 4. Discussion

Strawberry is an important fruit crop and is mainly consumed in its fresh form. Although strawberry production is increasing worldwide, it is infected by several fungal pathogens [39,40]. To reduce the losses caused by pathogenic fungi, farmers mainly rely on fungicides, which are reported to destroy the ozone layer [41,42]. Additionally, there have been several reports about resistance against fungicides [43]. Recently, the use of bio-fungicides to protect strawberry crops from pathogenic fungal infections has been increasing [44]. The current study was designed to better understand the fungal disease complex on the strawberry crop in China and to establish alternative methods for controlling the fungi. Transcriptome analysis of *Curvularia trifolii* in response to α-solanine treatment was performed. Earlier studies reported *Macrophomina phaseolina* and *Fusarium oxysporum* as the main fungi infecting strawberry crops [39,45]. Here, we report *C. trifolii* as a main causal agent of leaf spot disease in strawberry. In the present study, we compared the growth of *Curvularia* after α-Solanine treatment, which is a well-known anti-microbial alkaloid. Interestingly, α-solanine treatment resulted in poor growth, as well as cracked and rough surfaces on *Curvularia* spores. Previously, α-solanine was shown to increase autophagy and oxidative damage at the cellular level [10]. One of the most important findings in the present study is the differential growth and abundance of fungal biomasses. Hence, it is direct evidence that α-solanine acts as an inhibitor of fungal growth.

In the present study, sequencing of *C. trifolii* resulted in a total of 24,213 transcripts, which covers more than 90% of previously reported genome data [26]. Transcriptomic data analysis of treated samples revealed that the number of down-regulated genes was higher (441) than the number of up-regulated genes (280). A similar trend was also observed for unigenes. The down-regulated genes included some of the major components of cellular metabolism. For example, *TRINITY_DN10004_c0_g1* was identified as a down-regulating factor that plays an integral role in the RNA transcription of nuclear DNA. However, several genes involved in DNA binding, telomeres, or DNA repair were up-regulated in the presence of α-solanine. Interference in the lipid metabolism pathway may result in deformities in cellular membrane development [46]. Indeed, it can result in severe oxidative stress in the pathogen’s cells. Our findings are in agreement with earlier reports on antifungal agents [47].

The change in the reduction–oxidation status of the cells is a primary response to any stress condition. Several redox enzymes were differentially expressed (down-regulated) in α-solanine-treated *Curvularia* cultures. In the present study, the highest number of genes belonging to oxidation–reduction reactions was predicted through GO enrichment analysis. These processes are mainly involved in the degradation of extracellular cellulose and defense against other microbes and plants [48,49]. Most of the known phyto-pathogenic fungi utilize reactive oxygen species (ROS) for cellular signaling and interaction with plants. A higher level of gene expression related to ROS is plausible as it can be used to counter the effect of stress induced by α-solanine. It is well established that NADPH oxidases (Nox) encoded by fungi have three different sub-families and are primarily required for cellular growth, germination of ascospores, and sexual fruiting body development [50,51,52]. Additionally, suppression or mutation of NADPH results in cellular degeneration, loss of differentiation, increased fertility defects, and the immobilization of nutrients required for cellular growth [53]. Our data regarding poor fungal growth in the presence of α-solanine might be attributed to a higher level of ROS activation. It can be hypothesized that alkaloids such as α-solanine can inhibit hyphae and spore growth, and in return there is a burst of ROS in fungi to counter the external stress.

Fungal infection in plants can change the metabolome and microbiome profiles of the host [21]. Studies on the interaction of *Zymoseptoria tritici* with Chinese spring wheat and endophytic fungi with horseradish have shown the suppression of metabolic pathways including derivatives of amino acids, flavonoids, and phospholipids by the fungi [21,54]. Nonetheless, very few studies have been conducted on the metabolomic profile of specific fungi. The experiments conducted on the metabolic profile of filamentous fungi revealed that metabolites related to oxidative stress and phospholipid regulation play an important role in pathogenicity [55]. Our investigation of metabolites in the presence of antifungal components is important due to their unique biological activities. Using UHPLC-MS, we identified the differential expression of metabolites in *Curvularia*. Among the detected metabolites, a wide range of lipid biosynthesis-related pathways were identified. Interestingly, the accumulation of organo-oxygen compounds clearly indicates the anti-microbial effect of α-solanine. It has already been reported that secondary metabolites such as organo-oxygen compounds slow the growth of microorganisms, including fungi and bacteria. The differential expression of lipid metabolites or fatty acid derivatives is plausible since RNA-Seq data also support the significant changes in α-solanine-treated samples. Earlier studies also suggested that change in the lipid profile and membrane structure of pathogenic fungi is an indication of oxidative stress [56]. Our findings collectively indicate the efficacy of using α-solanine to retard the growth of pathogenic fungi by affecting genes related to lipid biosynthesis and oxidative stress pathways, thus altering secondary metabolites.

## 5. Conclusions

The present study concluded that treatment with α-solanine inhibited the growth and spore development of the *Curvularia trifolii* fungi isolated from strawberry. Transcriptomic analysis revealed significantly different profiles of gene expression in α-solanine-treated samples. Similarly, a differential profile of metabolites was identified in the control and treated fungi. The expression of genes related to the lipid biosynthesis pathway and lipid metabolites was altered in response to treatment with α-solanine. These findings indicated that α-solanine treatment had a cumulative impact on fungi related to lipid biosynthesis. Lipid biosynthesis stress is highly associated with oxidative stress in cells. The up-regulation of genes related to reactive oxygen species shows that α-solanine causes oxidative stress in Curvularia that leads to growth inhibition, thus indicating it could be potentially used as an alternative to chemical fungicides. The use of natural inhibitors against viral, bacterial, and fungal pathogens can present a more eco-friendly option and may even extend to their wider potential application as a new form of biopesticide.

## Figures and Tables

**Table 1:** Total number of clean reads, their size, and the corresponding length.

Sample ID	A1	A2	A3	B1	B2	B3
Number of reads (Millions)	42	48	44	44	44	48
Average read size	144	142	146	143	145	142
Total length	6,081,757,990	6,900,038,202	6,453,851,237	6,394,567,695	6,425,063,616	6,929,690,377
GC%	52.56	52.36	53.26	51.56	52.3	52.21
Q30%	92	92.18	91.72	91.9	92.31	92.32

## Data Availability

The sequence data generated in this study have been deposited in the SRA database of the NCBI under Project No. PRJNA885213.

## Supplementary Materials

The following supporting information can be downloaded at: https://www.mdpi.com/article/10.3390/life13020450/s1, Table S1. RNA-Seq data of α-solanine-treated *Curvularia* fungi isolated from strawberry fruit infected with leaf spot disease; Figure S1.The spore germination of *Curvularia trifolii* isolated from strawberry. A—control. B—treated with α-solanine (5 mg/mL). The α-solanine-treated samples showed poor growth, deformed cells, and very poor spore growth. Figure S2. Sequence length distribution in control (A) and treated fungi (B) samples; Figure S3. Similarity distribution of transcripts (A) and unigenes (B) revealed by a BLAST search in the non-redundant database; Figure S4. Venn diagram of totally differentially expressed unigenes in control (A) and treated (B) *Curvularia*.
