# Supplementary material for: Potential of Alpha-(α)-Solanine as a Natural Inhibitor of Fungus Causing Leaf Spot Disease in Strawberry
Source: Life (Basel). 2023 Feb 6;13(2):450. doi: 10.3390/life13020450 (PMC9961337; doi:10.3390/life13020450)
Supplement: Supplementary file 1 [file life-13-00450-s001.zip › life-2075351-supplementary.pdf]

Table S1. RNA-Seq data of α-solanine treated Curvularia fungi isolated from leaf spot disease of strawberry fruit.

Type	Control		α-solanine treated fungi	
	Unigenes	Transcripts	Unigenes	Transcirpts
Total number	18344	29058	17856	24213
Total base	24657575	54052701	24427313	38944986
Largest length (bp)	16387	16387	16387	16387
Smallest length (bp)	201	201	201	201
Average length (bp)	1344.18	1860.17	1368.02	1608.43
N50 length (bp)	2449	3245	2453	2789
E90N50 length (bp)	3072	2894	3072	3012
Mean mapped percent (%)	74.174	78.052	73.577	78.338
GC percent (%)	51.78	52.22	51.8	52.05
TransRate score	0.20653	0.17794	0.22352	0.23528

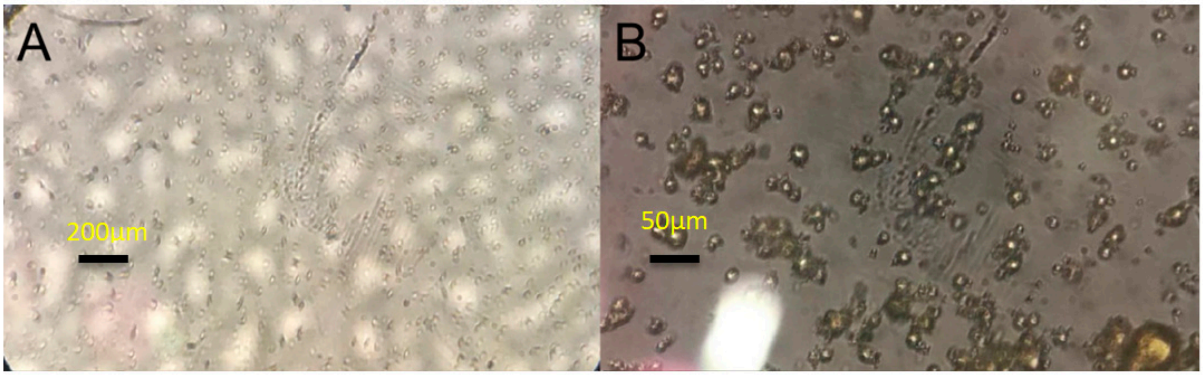

Figure S1. The spore germination of *CurvulariaCurvularia trifolii* isolated from strawberry. A- Control B- Treated with α-solanine (5mg/ml). The α-solanine treated samples have poor growth, deformed cells and very poor spore growth.

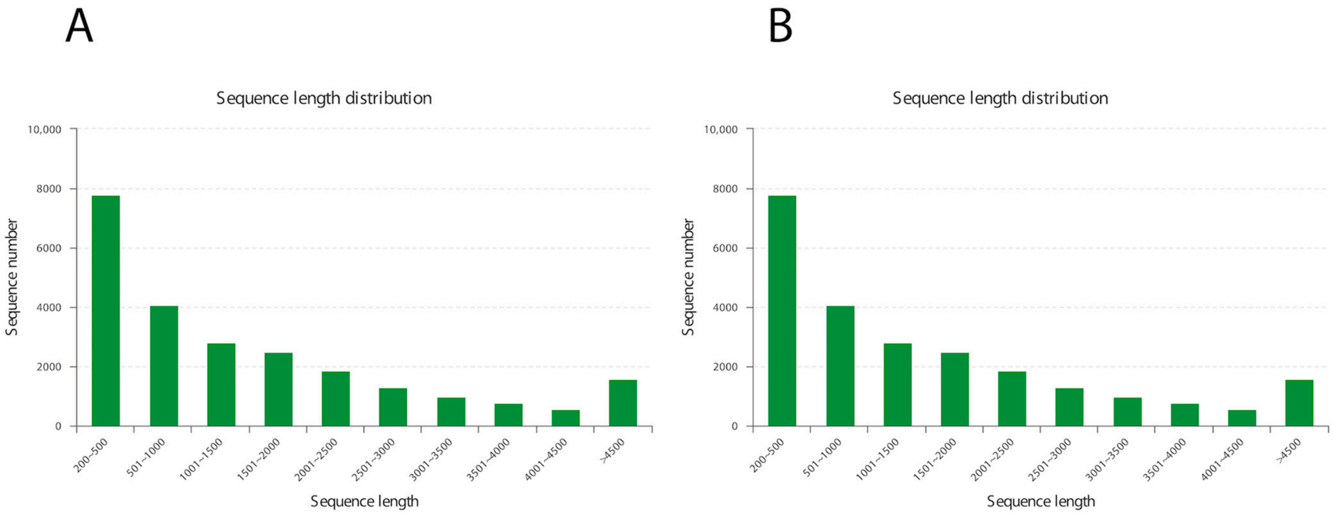

Figure S2. Sequence length distribution in control (A) and treated fungi (B) samples.

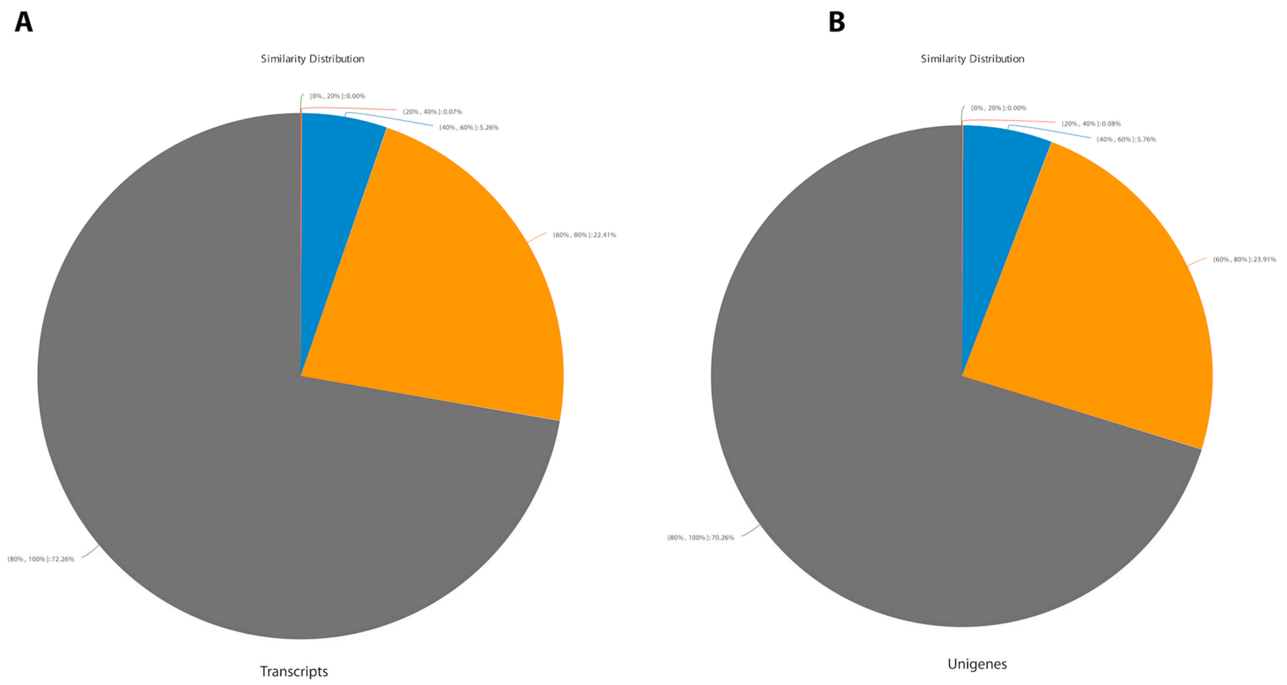

Figure S3. Similarity distribution of transcripts (A) and unigenes (B) revealed by BLAST search in non-redundant database.

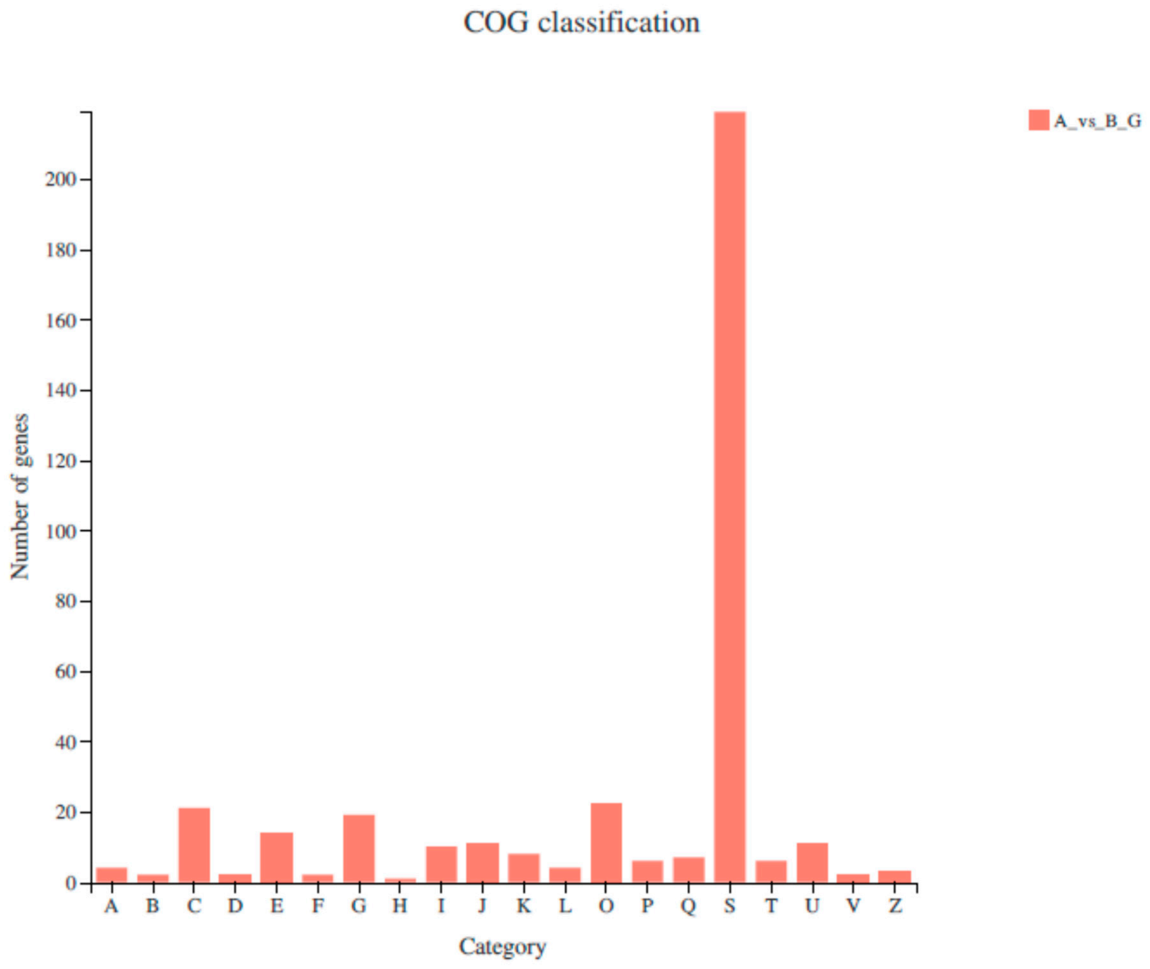

Figure S4. Distribution of total differentially expressed unigenes in control (A) and treated (B) *Curvularia*.
